# New Paradigms in the Evaluation of Diastolic Function by Cardiac Magnetic Resonance Imaging in Aortic Valvopathy

**DOI:** 10.36660/abc.20190903

**Published:** 2020-02

**Authors:** Vera Maria Cury Salemi, Marcelo Dantas Tavares de Melo, José de Arimatéia Batista Araujo Filho

**Affiliations:** 1 Instituto do Coração (InCor) do Hospital das Clínicas da Faculdade de Medicina da Universidade de São Paulo, São Paulo, SP - Brazil; 2 Universidade Federal da Paraíba, João Pessoa, PB - Brazil

**Keywords:** Echocardiography, Aortic Stenosis, Diastolic Function, Cardiac Magnetic Resonance, Aortic Valvopathy

The noninvasive analysis of left ventricular diastolic function is a challenge in clinical practice. Estimation of the time constant of isovolumetric relaxation - or Tau constant - is the best and most established parameter for ventricular diastolic function analysis. However, this measure is obtained through an invasive assessment and it is very difficult to acquire.^1^ In clinical practice, echocardiography is considered a key tool, among all the complementary exams that can be used to evaluate diastolic function, validated in relation to the pressure-volume curves by catheterization, restricting the invasive evaluation to be used in exceptional cases.^2^ Thus, echocardiography remains the first-line noninvasive method, providing data on early diastolic dysfunction through indices that reflect relaxation, compliance, and also indirect measurements of ventricular filling pressures.

The latest recommendations for diastolic dysfunction assessment by echocardiography basically include analysis of the left ventricle without structural changes and preserved ejection fraction, as well as a flowchart for cases of left ventricular ejection fraction reduction/structural change.^5^ However, the classification of the degree of diastolic dysfunction and its application in the therapeutic management of valvular heart disease remains controversial.^3,4^ Moreover, aortic stenosis may be associated with mitral annulus calcification, which may lead to a reduction of the mitral orifice area, with an increase in early transmitral diastolic velocity (E), while the Doppler lateral mitral annulus velocity (e’) may be reduced due to limitation of the posterior cusp excursion, which may lead to an artificial increase in the E/e’ ratio. In aortic regurgitation, however, the aortic reflux jet may interfere with the mitral flow, which, when significant, can lead to a restrictive ventricular filling pattern; however, the accuracy of E/e’ ratio is questionable.^5^

The present study proposes the analysis of diastolic function by analyzing left ventricular (LV) longitudinal movement, quantified by cardiac magnetic resonance imaging (cMRI).^6^ The study population consists of three groups according to hemodynamic stress: aortic stenosis (significant afterload increase), aortic regurgitation (consistent preload increase), both valvopathies compared with a healthy group. Both valvopathies comprised a comparative group with the controls. This study demonstrated that the LV longitudinal movement analysis was reduced in patients with aortic valve disease when compared to the control group, and that among patients with valvular disease, patients with aortic regurgitation had lower values than those with stenosis.

The cMRI is an attractive imaging modality capable of providing morphological, functional, perfusion and tissue characterization data in a single examination, with irrefutable spatial resolution. In the evaluation of diastolic function, recent techniques have shown encouraging results, especially those based on the myocardial strain assessment through feature tracking. However, the need for improvements in image postprocessing and acquisition times, together with the limited availability and relatively high cost of the employed software, limit a broader use of these relatively new technologies in clinical practice.^7^ It is in this context that the elegant study by Ribeiro et al.^6^ is located and has its greatest strength: in the cost-benefit of four variables obtained simply and quickly in the four-chamber view for indirect evaluation of diastolic dysfunction, without the need for additional specific sequences or the use of specific software. However, before proposing the incorporation of this technique into clinical routine, some important methodological considerations are necessary. We know that the ventricular longitudinal shortening measured by cMRI is classically related to ventricular systolic function and, more recently, also to diastolic dysfunction measured by echocardiography, even in patients with preserved ejection fraction.^8-10^

However, the authors did not directly correlate the ventricular longitudinal movement variables with Doppler echocardiographic variables, data with broad and widespread importance in the noninvasive investigation of diastolic dysfunction, or with invasive variables to validate these measurements.^10^ Although the authors assumed this fact as an important limitation of the study, this absence makes it impossible to estimate the accuracy, sensitivity, specificity and predictive values of this methodology. Furthermore, the lack of interobserver agreement assessment and internal and external validation, associated with the fact that the case group consisted of a very heterogeneous population (stenosis and valve regurgitation), require that these results be interpreted with extreme caution and cannot be extrapolated to other samples or distinct cardiomyopathies before being clinically validated. Other relevant data is consensually accepted that patients with severe aortic valve disease, either stenosis or regurgitation, already showing morphological alteration and ventricular compliance, are symptomatic. Patients with advanced aortic valvopathy have higher ventricular mass and reduced ejection fraction, when compared with the healthy group. In this context, measures of systolic and diastolic function can be expected to differ from those of the healthy control group.

As a suggestion for future studies, the inclusion of other variables, easily obtained in the basic cMRI sequences, such as left atrial volume (a chronic marker of diastolic dysfunction and cardiovascular risk) or the presence of late LV enhancement (indicator of fibrosis as a potential substrate for myocardial impairment) would certainly add important data to the debate on the role of cMRI in LV diastolic dysfunction. Moreover, it would be interesting to perform its validation with methods that are clinically used as invasive measures of the Tau constant, or through echocardiography parameters, which are validated measures for the analysis of diastolic function in different heart diseases.
